# Molecular Detection and Phylogenetic Analysis of Tick-Borne Encephalitis Virus from Ticks Collected from Cattle in Kyrgyzstan, 2023

**DOI:** 10.3390/v16010107

**Published:** 2024-01-11

**Authors:** Haneul Jung, Chi-Hwan Choi, Minji Lee, Seong-Yoon Kim, Bekbolsun Aknazarov, Rysbek Nyrgaziev, Nurzina Atabekova, Elmurat Jetigenov, Yoon-Seok Chung, Hee-Il Lee

**Affiliations:** 1 Division of Vectors and Parasitic Diseases, Korea Disease Control and Prevention Agency (KDCA), Cheongju 28159, Republic of Korea; sky123664@korea.kr (H.J.); gunbo0402@korea.kr (S.-Y.K.); 2 Division of High-Risk Pathogens, Korea Disease Control and Prevention Agency (KDCA), Cheongju 28159, Republic of Korea; chihwanchoi@korea.kr (C.-H.C.); mjlee4245@korea.kr (M.L.); rollstone93@korea.kr (Y.-S.C.); 3 Faculty of Veterinary Medicine, Kyrgyz National Agrarian University Named after K. I. Skryabin, Bishkek 720005, Kyrgyzstan; aknazaroov-61@mail.ru (B.A.); rysbekn@mail.ru (R.N.); atbn.7@mail.ru (N.A.); agetigen@mail.ru (E.J.)

**Keywords:** Kyrgyzstan, tick-borne encephalitis virus, next-generation sequencing

## Abstract

Ticks are important vectors of the tick-borne encephalitis virus (TBEV). In Kyrgyzstan, the livestock farming trade and nomadic lifestyle enable tick-borne diseases to be imported from neighboring countries, but there are few relevant studies. In this study, we collected 40 ticks from cattle in Kyrgyzstan. Molecular marker analysis identified the ticks as *Ixodes persulcatus* (97.5%; *n* = 39) and *Haemaphysalis punctata* (2.5%; *n* = 1). Real-time PCR screening revealed two ticks to be positive for TBEV, but only one tick was amplified using nested PCR targeting the TBEV envelope (E) and non-structure 5 (NS5) gene. The obtained sequences belonged to the TBEV Siberian subtype and phylogenetic tree analysis results confirmed that the virus was related to the Bosnia strain. We also performed next-generation sequencing, which confirmed the TBEV Siberian subtype. Continuous research and surveillance of TBEV in Kyrgyzstan are required to provide further information on tick-borne diseases.

## 1. Introduction

Ticks are vectors for transmitting viral pathogens including tick-borne encephalitis virus (TBEV), Crimean–Congo hemorrhagic fever virus (CCHFV), Powassan virus, and deer tick virus [1]. The tick-borne encephalitis virus (TBEV) is a single-stranded, positive-sense RNA virus of the genus *Flavivirus* [2]. It belongs to the mammal-associated group [3] and is prevalent across the Eurasia continent, including Europe, Russia, Turkey, Kazakhstan, Kyrgyzstan, Mongolia, China, Japan, etc. [4,5]. Tick-borne encephalitis (TBE) mainly causes neurological symptoms, such as meningitis, encephalitis, and myelitis, accompanied by fever. However, there is no specific antiviral treatment for TBE [6].

TBEV is mainly divided into three subtypes: European (TBEV-Eu), Far-Eastern (TBEV-Fe), and Siberian (TBEV-Sib). At least 14 species of ticks can act as a vector for TBEV, but there are some specific vectors and virus subtypes. *Ixodes ricinus* is an important vector of TBEV-Eu and *I. persulcatus* is the principal vector of TBEV-Fe and TBEV-Sib [1,7]. TBEV-Eu is mainly distributed in Central Europe and has the lowest fatality rate (1–2%) among the three subtypes. TBEV-Fe, which is found in far-eastern Asia and central/eastern Siberia, has a high fatality rate of 20–40%, and TBEV-Sib, which is distributed in Siberia, has a moderately low fatality rate of 6–8% [3,8]. Recently, new TBEV subtypes, including Baikalian (TBEV-Bkl) and Himalayan (TBEV-Him), have been reported in Russia and China, respectively [9,10].

Reservoir hosts of TBEV are small rodents (genera *Myodes* and *Apodemus*), and TBEV can be transmitted from infected ticks to vertebrate hosts during feeding [11]. Horizontal transmission by co-feeding can also transmit the virus, but the role of accidental hosts, such as large vertebrates and humans, in virus transmission between ticks is considered insignificant [12,13]. Humans can be infected with TBEV through tick bites or ingesting unpasteurized raw milk and dairy products from infected animals [14,15]. In addition, the movement of migratory birds is also considered to be an important TBEV transmission route [16,17].

The World Health Organization (WHO) has reported 10,000–12,000 clinical cases of TBE annually. Between 2016 and 2020, approximately 3000 cases were registered in EU/EEA (European Union and European Economic Area) countries annually [18] and 1500–2000 cases were recorded in Russia over the last six years (2013–2018) [19]. In Kazakhstan, about 363 cases of TBE were reported in the last ten years (2011–2020) [20]. Tick-borne diseases are also suspected to be endemic in other countries in Central Asia, including Kyrgyzstan, Tajikistan, Turkmenistan, and Uzbekistan [21], but few studies have been conducted in these areas, except for one study where TBEV was found in Kyrgyzstan ticks [22].

The Korea Disease Control and Prevention Agency has cooperated with Kyrgyz National Agrarian University to conduct national tick surveillance since 2020. In the only previous study in Kyrgyzstan, the TBEV-Sib strain, which is related to the Novosibirsk strain, was confirmed in ticks and rodents from Ala-Archa National Nature Park [22]. However, since then, no vector surveillance related to tick-borne viral pathogens, including TBEV and CCHFV, has been performed in Kyrgyzstan. In this study, we aimed to investigate the prevalence of TBEV and CCHFV in ticks in Kyrgyzstan and to better understand the transmission and distribution of tick-borne pathogens.

## 2. Materials and Methods

### 2.1. Tick Collection

**Figure 1:**
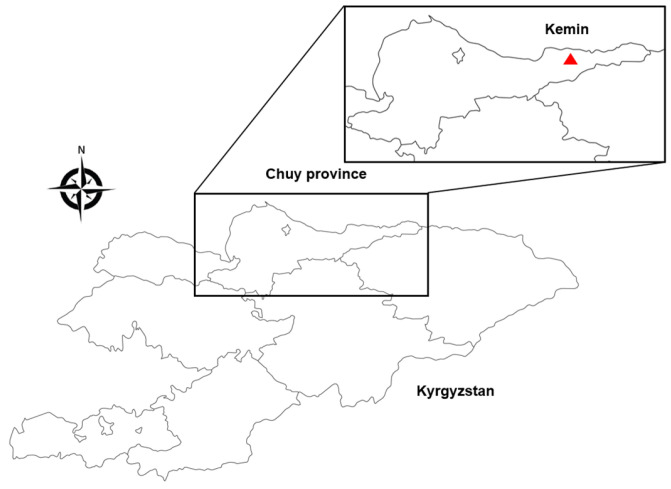
Map for study site in the Kemin region (red triangle), Chuy province, Kyrgyzstan. Adapted from [25].

Ticks were detached from cattle grazing in a pasture in the Kemin region in Chuy province of Kyrgyzstan in May 2023 (Figure 1). After collection, ticks were stored in a 50 mL tick collection tube (SPL, Seoul, Republic of Korea; patent no. 10-0925882) [23] with wet tissue paper to keep the ticks alive until testing. Their species and developmental stage were identified under a dissection microscope (Olympus, Tokyo, Japan) based on a morphological classification key [24].

### 2.2. Tick Sample Preparation

Ticks were individually homogenized in a Reinforced Bead tube (Zirconia 3 mm, Clear tube) in the Clear-S^TM^ Total RNA Extraction Kit (Invirustech, Gwangju, Republic of Korea) using Universal Transport Medium (Copan, Brescia, Italy) and Precellys 24 homogenizer (Bertin Technologies, Montigny-le-Bretonneux, France). After centrifuging the homogenate at 13,000 rpm for 10 min, the supernatant was divided into two parts; RNA was extracted from one half according to the manufacturer’s instructions, and the other half was reserved for next-generation sequencing (NGS) analysis. The RNA Lysis D(RLD) buffer in the RNA extraction kit was added to the debris in the bead tube, and DNA was extracted for species identification using molecular markers.

### 2.3. Molecular Identification of Tick Species

Tick species were confirmed according to morphological classification under a dissection microscope and on a chilling table. The molecular markers used to identify tick species were a 710 bp fragment of the mitochondrial cytochrome c oxidase subunit Ⅰ gene (COⅠ) [26], a 380 bp fragment of 12S ribosomal DNA (rDNA) [27], and a 395 bp fragment of 16S rDNA [28]. For the polymerase chain reaction (PCR), 5 μL of DNA was amplified using the AccuPower PCR PreMix (Bioneer, Daejeon, Republic of Korea) and the primers listed in Table 1.

### 2.4. Molecular Detection of TBEV and CCHFV

Molecular detection of tick-borne viral pathogens in collected ticks was performed using the PowerChek^TM^ TBEV Multiplex Real-time PCR Kit (European/Far-Eastern/Siberian) (KogeneBiotech, Seoul, Republic of Korea) and the LiliF^®^ CCHFV Real-time RT-PCR Kit (iNtRON Biotechnology, Gyeonggi-do, Republic of Korea). The RNA from all positive samples identified in real-time PCR screening were reverse-transcribed to cDNA using the RNA to cDNA EcoDry^TM^ Premix (Random Hexamers) (Takara Korea Biomedical Inc., Seoul, Republic of Korea). To examine for the presence of the TBEV envelope (E) [29] and non-structure 5 (NS5) genes [30], nested PCR was performed using the AccuPower PCR PreMix (Bioneer, Daejeon, Republic of Korea) and the primers listed in Table 1. AMPLIRUN^®^ TICK-BORNE ENCEPHALITIS VIRUS RNA CONTROL (MBC045-R) (Vircell, Granada, Spain) and CCHFV synthetic RNA were used as the positive control by the authors, and a negative control was used to identify contamination. The amplified PCR products were confirmed using the QIAxcel capillary electrophoresis system (Qiagen, Hilden, Germany).

### 2.5. Nucleotide Sequencing and Phylogenetic Analysis

The PCR products from the tick species identification experiments used COⅠ, 12S rDNA, and 16S rDNA as molecular markers, and the verified TBEV E and NS5 genes were sequenced. Nucleotide sequence homology searches were performed using the National Center for Biotechnology Information (NCBI, Bethesda, MD, USA) BLAST network service. The phylogenetic tree was constructed using the maximum likelihood (ML) method with the Kimura 2-parameter model in MEGA 11. All reference sequences used for phylogenetic analysis were downloaded from GenBank.

### 2.6. NGS Analysis

RNA was extracted from tick homogenates using the QIAamp^®^ Viral RNA mini kit (Qiagen, Hilden, Germany), followed by cDNA synthesis using the RNA Prep with Enrichment kit (Illumina, San Diego, CA, USA). The MiSeq libraries were prepared using the Illumina Viral Surveillance Panel (Illumina, San Diego, CA, USA), which can detect 66 critical viruses, including Omsk hemorrhagic fever virus, TBEV, and CCHFV, with a hybridization probe-capture-based approach. NGS was performed on the MiSeq platform using paired-end sequencing with 150 bp × 2 formats.

The obtained sequences were analyzed using CLC Genomics Workbench 20 (Qiagen, Hilden, Germany), and reference mapping was performed against the reference genome (GenBank accession number: NC_001672), resulting in the generation of consensus genome sequences. Phylogenetic analysis was conducted using the Nextstrain CLI 7.2.0 analysis pipeline with metadata and sequence data from the previously described TBEV Nextstrain database [31]. Evolutionary rates were determined based on a previously described molecular clock rate of 5.96 × 10^−5^ substitutions per site per year with a standard deviation of 6.6 × 10^−6^ [32]. Furthermore, TBEV Analyzer was utilized for the subtype classification of the genome [33].

## 3. Results

### 3.1. Tick Identification

**Figure 2:**
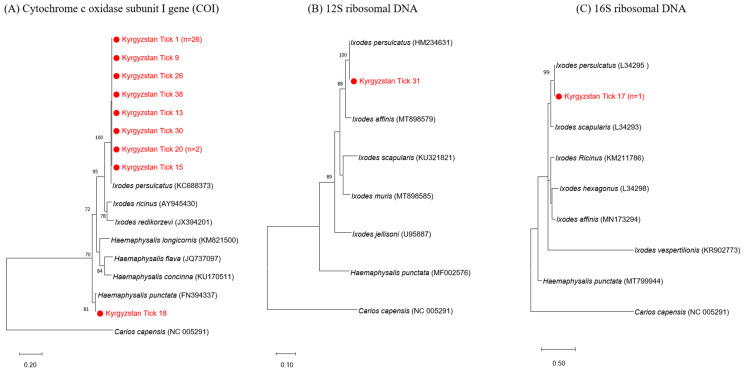
Phylogenetic analysis for tick identification based on the partial nucleotide sequences of (**A**) COⅠ, (**B**) 12S rDNA, and (**C**) 16S rDNA. The maximum likelihood (ML) method was used with the Kimura 2-parameter model. The number on the branches indicates bootstrap percentages based on 1000 replications. The cut-off value for the consensus tree was 70%. The sequences identified in this study are indicated by red dots. The number of sequences (*n*) with a corresponding identical sequence is shown if the sequence was detected in more than one case. The bar indicates the number of nucleotide substitutions per site.

A total of 40 partially or fully engorged adult tick specimens were identified as belonging to two genera of Metastigmata: Ixodid ticks. Molecular analysis confirmed the proportion of tick species and identified the ticks as *Ixodes persulcatus* (97.5%; *n* = 39) and *Haemaphysalis punctata* (2.5%; *n* = 1). Additionally, 36 *I. persulcatus* sequence and 1 *H. punctata* sequence were identified by the COⅠ gene (Figure 2, panel A). One additional *I. persulcatus* sequence was confirmed by the 12S rDNA (Figure 2, panel B). The remaining two specimens were confirmed as *I. persulcatus* through the 16S rDNA gene (Figure 2, panel C).

Furthermore, phylogenetic analysis of the tick species showed that the representative sequence of each tick species was clustered with the related *Ixodes* spp. and *Haemaphysalis* spp. reference sequences of COⅠ, 12S rDNA, and 16S rDNA.

### 3.2. TBEV and CCHFV Detection

**Figure 3:**
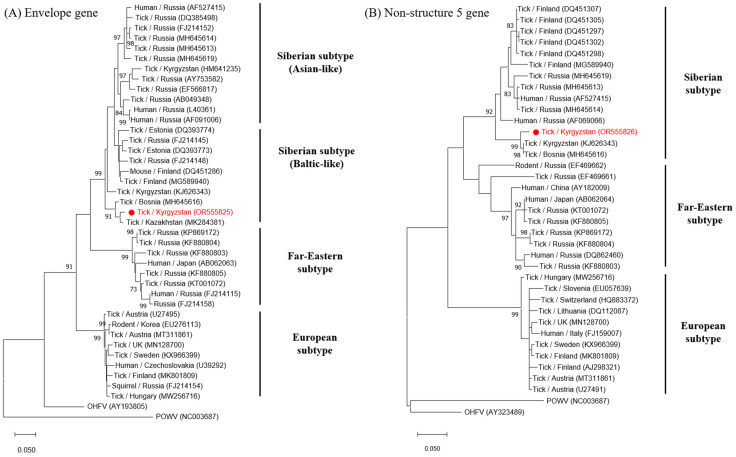
Phylogenetic analysis of TBEV (**A**) E and (**B**) NS5 gene-positive sequences. The maximum likelihood (ML) method was used with the Kimura 2-parameter model. The number on the branches indicates bootstrap percentages based on 1000 replications. The cut-off value for the consensus tree was 70%. The sequences identified in this study are indicated by red dots. The bar indicates the number of nucleotide substitutions per site.

Among the 40 ticks, CCHFV was not detected, whereas 2 *I. persulcatus* (5.0%) were positive for TBEV, as determined by real-time PCR screening. However, in nested PCR, only one positive sample was amplified. The amplified TBEV E (477 bp) and NS5 genes (252 bp) of the positive sample were sequenced and compared with other TBEV sequences in the GenBank database. The TBEV E gene sequence in this study showed high similarity to the MK284381 (Kazakhstan, 98.01%) and MH645616 (Bosnia, 97.13%) sequences of the Baltic-like strain of TBEV-Sib (Figure 3A). The NS5 gene sequence showed high similarity to the KJ626343 (Kyrgyzstan, 98.10%) and MH645616 (Bosnia, 98.10%) sequences of TBEV-Sib (Figure 3B). The acquired TBEV E (OR555825) and NS5 gene (OR555826) sequences were submitted to NCBI GenBank.

### 3.3. NGS Analysis

**Figure 4:**
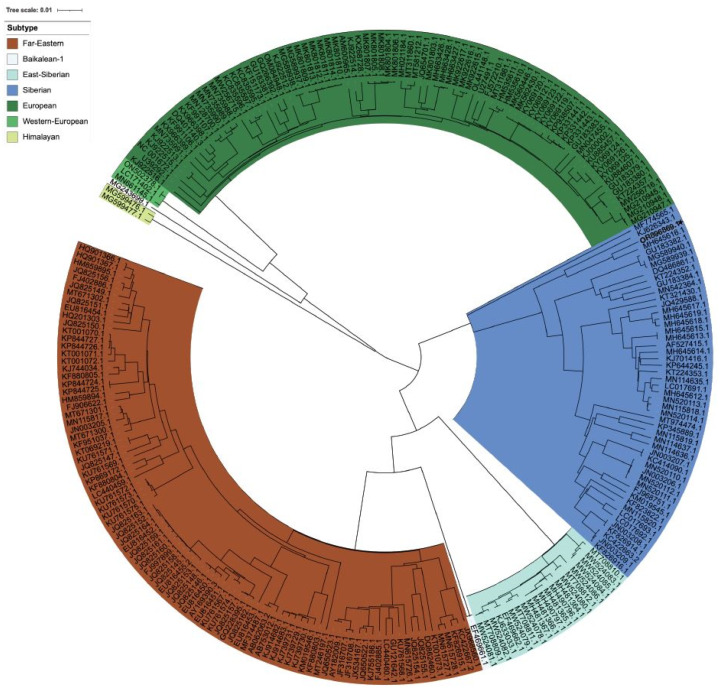
Radial phylogenetic tree of TBEV sequences. The radial phylogenetic tree was constructed using IQ-tree v2.1.2 and the maximum likelihood (ML) with the General Time Reversible (GTR) model, embedded in Nextstrain CLI 7.2.0. The tree was divided into seven subtypes (Far Eastern, Baikalean-1, East Siberian, Siberian, European, Western European, and Himalayan), with labels indicating the corresponding GenBank accession numbers of the genomes. Within the tree, OR896869 was classified as the Siberian subtype, revealing a close relationship with the MH645616.1 (Bosnia) and KJ626343.1 (Kyrgyzstan) genome sequences.

Among the 66 critical viruses, only TBEV was detected by Illumina Viral Surveillance Panel (Illumina, San Diego, CA, USA). In MiSeq NGS analysis of TBEV, a total of 1,442,079 reads were generated, providing a genome depth of approximately 63,442 X, with a GC content of 53.8% and a genome length of 11,141 bp. The NGS sequence (OR896869) of this study was submitted to NCBI GenBank. Phylogenetic analysis conducted using Nextstrain revealed that the closest genome was that of the Bosnia strain (MH645616) within TBEV-Sib (Figure 4). Additionally, genome subtype classification using TBEV Analyzer online confirmed TBEV-Sib. FastANI analysis of 254 sequences resulted in average nucleotide identity scores, validating the identities of MH645616.1 (Bosnia, 97.3%) and KJ626343.1 (Kyrgyzstan, 93.9%).

## 4. Discussion

We identified two tick species, *I. persulcatus* (*n* = 39) and *H. punctata* (*n* = 1), and two TBEV-positive ticks were identified by real-time PCR screening, but only one tick was amplified for identification of the TBEV E and NS5 genes by nested PCR and NGS analysis.

Based on phylogenetic analysis, TBEV is systematically classified into three major subtypes (TBEV-Eu, TBEV-Fe, and TBEV-Sib) [1,7]. Of these, FJ214152 is a representative sequence of TBEV-Sib (Asian-like) isolated from Russia. MG589940, KF880803, and MT311861 are representative of TBEV-Sib (Baltic-like), TBEV-Fe, and TBEV-Eu isolated from Finland, Russia, and Austria, respectively (Figure 3). In our study, the amplified sequences of TBEV based on the partial E (OR555825) and NS5 (OR555826) genes were classified as the Siberian subtype.

TBEV-Sib has also been detected in Kazakhstan and China, which share a border with Kyrgyzstan. However, there are no studies on TBEV in ticks found in Uzbekistan, Tajikistan, and Turkmenistan. In Kazakhstan, 48 pools were confirmed for TBEV-Sib among 1737 ticks (*I. persulcatus*, *H. punctata,* and *Dermacentor marginatus*), and the minimum infection rate (MIR) was 4.4% in Targer, 2.8% in Tekeli, and 1.1% in Yenbekshikazakh in the Almaty province [34]. Moreover, in the Xinjiang Uygur Autonomous Region in the northwest of China, TBEV-Sib and TBEV-Fe were isolated from *I. persulcatus* (MIR = 14.3–47.7%) and *D. silvarum* (MIR = 0.01–1.67%) [35,36,37]. Thus, TBEV-Sib is prevalent in Central Asia, and the main vector is *I. persulcatus*, but other ticks can also transmit TBEV-Sib [1,7].

In a study in Kyrgyzstan [22], TBEV-Sib was detected in ticks (six positive pools among 222 Ixodid ticks). A limitation of our study is the small sample size of ticks compared to that of the previous study. However, the proportion of TBEV-infected ticks was similar to that of the previous study (approximately 2.5%, 1 positive tick among 40 ticks). By contrast, the TBEV E gene sequences from the previous study (HM641235) were related to the Novosibirsk strain, which originated from Russia [22]. However, the TBEV E gene sequence (OR555825) identified in this study was associated with the Bosnia strain (MH645616), which originated from the Balkan Peninsula [38]. The TBEV NS5 gene sequence (OR555826), which was the first to be identified in Kyrgyzstan, was also confirmed to be related to the Bosnia strain (MH645616). The previous study was conducted 16 years ago, but TBEV-Sib is still in circulation in Kyrgyzstan and exhibits genetic variation.

Studying tick-borne pathogens poses significant challenges, primarily owing to difficulties in gene amplification, especially concerning genes with a low viral load. These challenges not only hinder the production of sequencing data but also impact the reproducibility of results. In this study, obtaining gene fragments from tick homogenate samples using both real-time PCR and conventional PCR methods was crucial. Furthermore, our research team employed a specific target enrichment method to analyze the entire 11,141 bp TBEV genome from tick fluid homogenate, achieving 100% coverage. This analysis revealed that the sequence (OR896869) had the highest homology with that of the Bosnia strain (MH645616). This result is consistent with those of the TBEV E and NS5 gene phylogenetic tree analyses and can provide a scientific basis for the location of the sequence. The specific target capture enrichment method utilized in this study represents a pioneering advancement in analyzing small amounts of genetic material within ticks. It may be a crucial technique for genomic analysis of disease vector insects like ticks, offering invaluable insights into the transmission of infectious diseases.

Kyrgyzstan is a landlocked country located in the mountainous terrain of eastern Central Asia and in the center of the Silk Road, which has been a trade route for thousands of years. Kyrgyzstan remains a vital international hub for linking regional communities and the movement of many goods [21,39]. The pursuit of livestock farming (mainly of goats, sheep, and horses) as the main economic activity in Kyrgyzstan contributes to the nomadic lifestyle of the people [40,41]. Owing to the rugged mountainous region, horses, mules, and donkeys are an important form of transportation [42]. These geographical and social cultural characteristics may facilitate the movement of ticks and increase the spread of tick-borne diseases.

Travel is one of the causes of tick distribution across countries. According to Public Health England’s Tick Surveillance Scheme in the United Kingdom (UK), 10 different tick species of six different genera have been identified on animals with a history of travel outside of the UK [43]. Therefore, ticks may move between countries via the Kyrgyzstan border, where there is relatively free movement of livestock and goods over land trade. Recently, the importation of ticks via travelers was confirmed in Brazil and Mexico [44,45]. As many travelers visit the Ala-Archa National Natural Park in Kyrgyzstan annually, there is a possibility of the introduction of ticks and tick-borne pathogens through people, including travelers. Nevertheless, there is a lack of research related to the spread of tick-borne diseases. In particular, TBEV has only been studied in the northern region of Kyrgyzstan, the main destination of travelers [22]. Investigations of TBEV in the southern region of Kyrgyzstan remain limited, and therefore further tick surveillance is needed to confirm the prevalence of TBEV.

In addition to TBEV, Crimean–Congo hemorrhagic fever virus (CCHFV), which causes a fatal tick-borne disease, is continuously being recorded in countries surrounding Kyrgyzstan. In the southern region of Kazakhstan, which shares a border with Kyrgyzstan, CCHFV was detected in ticks (*Hyalomma* spp. and *I. ricinus*) and livestock (cattle and sheep) [46,47]. CCHFV-infected ticks (*Hyalomma* spp. and *Dermacentor nuttalli*) were also recorded in the Xinjiang Uygur Autonomous Region of northwestern China [48,49], and CCHFV patients have been reported in Tajikistan [50,51]. This suggests that various tick-borne diseases, such as those caused by TBEV and CCHFV, can be transmitted across borders. Although CCHFV was not detected in this study, we still consider the possibility that CCHFV is able to be imported to Kyrgyzstan from neighboring countries. To date, there is insufficient research related to viral pathogens in ticks in Kyrgyzstan. More tick investigations and surveillance are required to better understand the distribution and spread of viral pathogens that are a risk factor for outbreaks and epidemics of multiple diseases in Kyrgyzstan.

In summary, a different strain of TBEV-Sib from those identified in previous studies was identified in Kyrgyzstan. Considering the geographical location and socio-cultural features of Kyrgyzstan, continuous research and surveillance of ticks should be conducted to understand the features of imported tick-borne diseases and the distribution of pathogens.

## Figures and Tables

**Table 1:** Primers used in this study.

Gene	Primer	Sequence (5′→3′)	PCR Condition	Reference
COⅠ	LCO1490	GGTCAACAAATCATAAAGATATTGG	95 °C, 5 min; 35 cycles (95 °C 1 min, 40 °C 1 min, 72 °C 30 s); 72 °C, 10 min	[26]
	HCO2198	TAAACTTCAGGGTGACCAAAAAATCA		
12S rDNA	12-SA1	AAACTAGGATTAGATACCCTATTAT	95 °C, 2 min; 28 cycles (95 °C 45 s, 52 °C 45 s, 72 °C 45 s); 72 °C, 10 min	[27]
	12-SB1	AAGAGCGACGGGCGATGTGT		
16S rDNA	16S-F	TTAAATTGCTGTRGTATT	94 °C, 5 min; 5 cycles (94 °C 30 s, 49 °C 30 s, 68 °C 30 s); 5 cycles (94 °C 30 s, 47 °C 30 s, 68 °C 30 s); 5 cycles (94 °C 30 s, 45 °C 30 s, 68 °C 30 s); 25 cycles (94 °C 30 s, 43 °C 30 s, 68 °C 30 s); 68 °C, 5 min	[28]
	16S-R2	CAACATCGAGGTCGCAAWCYA		
E	TBE913F	TGCACACAYYTGGAAAACAGGGA	94 °C, 5 min; 30 cycles (94 °C, 30 s; 52 °C, 30 s; 72 °C, 1 min); 72 °C, 5 min	[29]
	TBE1738R	TGGCCACTTTTCAGGTGGTACTTGGTTCC		
	TBE1192F	CAGAGTGATCGAGGCTGGGGYAA	94 °C, 2 min; 30 cycles (94 °C, 20 s; 62 °C, 10 s; 68 °C, 20 s); 70 °C, 5 min	
	TBE1669R	AACACTCCAGTCTGGTCTCCRAGGTTGTA		
NS5	FSM-1	GAGGCTGAACAACTGCACG	94 °C, 5 min; 35 cycles (96 °C 30 s, 40 °C 30 s, 68 °C 30 s); 72 °C, 5 min	[30]
	FSM-2	GAACACGTCCATTCCTGATCT		
	FSM-1i	ACGGAACGTGACAAGGCTAG	94 °C, 5 min; 30 cycles (96 °C 30 s, 40 °C 30 s, 68 °C 30 s); 72 °C, 5 min	
	FSM-2i	GCTTGTTACCATCTTTGGAG		

PCR—Polymerase chain reaction.

## Data Availability

The data presented in this study are available in the article.
